# Advanced age is an independent prognostic factor of disease progression in high-risk prostate cancer: results in 180 patients treated with robot-assisted radical prostatectomy and extended pelvic lymph node dissection in a tertiary referral center

**DOI:** 10.1007/s40520-023-02466-z

**Published:** 2023-06-19

**Authors:** Antonio Benito Porcaro, Alberto Bianchi, Sebastian Gallina, Andrea Panunzio, Emanuele Serafin, Giovanni Mazzucato, Rossella Orlando, Francesca Montanaro, Giulia Marafioti Patuzzo, Alberto Baielli, Francesco Artoni, Francesco Ditonno, Stefano Vidiri, Damiano D’Aietti, Filippo Migliorini, Riccardo Rizzetto, Alessandro Veccia, Alessandra Gozzo, Matteo Brunelli, Alessandro Tafuri, Maria Angela Cerruto, Alessandro Antonelli

**Affiliations:** 1 Department of Urology, University of Verona, Azienda Ospedaliera Universitaria Integrata Verona, Piazzale Stefani 1, 37126 Verona, Italy; 2 Department of Pathology, University of Verona, Azienda Ospedaliera Universitaria Integrata, Verona, Italy; 3 Department of Urology, Vito Fazzi Hospital, 73110 Lecce, Italy

**Keywords:** Prostate cancer, Intermediate-risk prostate cancer, Robot-assisted radical prostatectomy, Prognostic factors, Prognostic groups, Adverse pathology, Tumor upgrading, Tumor upstaging, Prostate cancer progression

## Abstract

**Objectives:**

This study aimed to assess more clinical and pathological factors associated with prostate cancer (PCa) progression in high-risk PCa patients treated primarily with robot-assisted radical prostatectomy (RARP) and extended pelvic lymph node dissection (ePLND) in a tertiary referral center.

**Materials and methods:**

In a period ranging from January 2013 to October 2020, RARP and ePLND were performed on 180 high-risk patients at Azienda Ospedaliera Universitaria Integrata of Verona (Italy). PCa progression was defined as biochemical recurrence/persistence and/or local recurrence and/or distant metastases. Statistical methods evaluated study endpoints, including Cox’s proportional hazards, Kaplan-Meyer survival curves, and binomial logistic regression models.

**Results:**

The median age of included patients was 66.5 [62–71] years. Disease progression occurred in 55 patients (30.6%), who were more likely to have advanced age, palpable tumors, and unfavorable pathologic features, including high tumor grade, stage, and pelvic lymph node invasion (PLNI). On multivariate analysis, PCa progression was predicted by advanced age (≥ 70 years) (HR = 2.183; 95% CI = 1.089–4377, *p* = 0.028), palpable tumors (HR = 3.113; 95% CI = 1.499–6.465), *p* = 0.002), and PLNI (HR = 2.945; 95% CI = 1.441–6.018, *p* = 0.003), which were associated with clinical standard factors defining high-risk PCa. Age had a negative prognostic impact on elderly patients, who were less likely to have palpable tumors but more likely to have high-grade tumors.

**Conclusions:**

High-risk PCa progression was independently predicted by advanced age, palpable tumors, and PLNI, which is associated with standard clinical prognostic factors. Consequently, with increasing age, the prognosis is worse in elderly patients, who represent an unfavorable age group that needs extensive counseling for appropriate and personalized management decisions.

## Introduction

Prostate cancer (PCa) is an epidemic issue due to the extensive opportunistic screening by prostate-specific antigen (PSA) in aging males [1, 2]. Prognosis is defined by classification systems whose aim is to group homogenous sets of patients [1–3]. Although the European Association of Urology (EAU) and the National Cancer Comprehensive National Network (NCCN) refer to D’Amico’s risk classes, they are not equivalent [1, 2]. According to the International Society of Urologic Pathology (ISUP) system, the high-risk category is defined by the combination of three main clinical prognostic factors which are: PSA > 20 ng/mL, palpability of prostate tumors (stage cT2-4) and high-grade tumors (ISUP 4/5). Moreover, the regional status of pelvic lymph nodes (cN0 vs cN1) is also considered by both systems [1, 2]. However, high-risk PCa is one of the most controversial prognostic categories because it differs in the two major referral systems and includes a pathologically nonhomogeneous set of patients [1, 2]. According to EAU, high-risk PCa includes localized and locally advanced diseases. The difference lies in the tumor stage (cT3/4 vs cT2c) and/or imaging findings suggesting pelvic lymph node involvement (cN1 vs cN0) [1].

On the other hand, according to the NCCN, the categories considered include high-risk and very high-risk PCa, with the latter associated with a more advanced clinical tumor stage (cT3/4) than the former (cT3). Notably, palpable tumors involving both lobes of the prostate are classified as cT2c, which is included in the intermediate-risk group according to the NCCN but in the high-risk group according to the EAU [1, 2]. Currently, the main recommendations for the treatment of localized high-risk PCa include radical prostatectomy or radiation therapy ± androgen deprivation therapy. Radical prostatectomy is most frequently performed by robot-assisted approach (RARP) associated with extended pelvic lymph node dissection (ePLND) for a selected set of patients, and as part of a potential multi-modal therapy or intensity-modulated radiation therapy (IMRT) in combination with long-term (two or three years) androgen deprivation therapy (ADT) [1, 2]. Notably, high-risk patients, when compared to the other risk classes, are at a higher risk of disease recurrence and progression after treatments. However, the prognosis is not uniformly poor after primary surgery because of a nonnegligible rate of organ-confined disease (26–31%) and a high rate of tumor downgrading between the biopsy ISUP grade and the ISUP grade group, as detected in the surgical specimen [1, 2]. To date, high-risk PCa remains a non-homogenous clinical category in terms of risk factors, which may combine different patterns and staging limitations, leading to down-staging and downgrading phenomena after surgery. However, pending the introduction of prognostic molecular markers, there is a need to improve standard clinical predictors of disease progression in high-risk PCa to reduce treatment-related regret. The latter is becoming increasingly critical in daily clinical practice for urologists and radiation oncologists [4]. This study aimed to assess more clinical and pathological factors of PCa progression in high-risk PCa patients treated primarily with RARP and ePLND in a tertiary referral center.

## Materials and methods

### Data collection, patient selection, evaluation of clinical parameters

Institutional Review Board approval was obtained from Azienda Ospedaliera Universitaria Integrata of Verona’s ethical committee. Informed consent for data analysis and publication was obtained for all subjects. Data were collected prospectively but evaluated retrospectively. Patients were classified into risk classes, according to EAU guidelines [1]. Between January 2013 and October 2020, 1143 patients underwent RARP, but complete follow-up was only available in 901 subjects because 242 patients were lost at the beginning or during follow-up. Of the 901 included patients, 180 belonged to the high-risk class. In all cases, PSA (ng/mL), age (years), body mass index (BMI; kg/m^2^), prostate volume (PV; mL), and percentage of biopsy-positive cores (BPC; %) were evaluated. Clinical staging was assessed by the 2017 version of the TNM system (8th edition) with clinical T stage referring only to digital rectal exam findings. Preoperative physical status was assessed using the American Society of Anesthesiologists (ASA) system [5]. RARP and ePLND were performed by experienced surgeons. Dissected lymph nodes were submitted in separate packages according to a standard anatomical template including external iliac, internal iliac and obturator, Marcille’s common iliac, and Cloquet’s nodal stations, bilaterally [6, 7].

### Evaluation of pathological parameters

Specimens were evaluated by a dedicated pathologist. Prostates were weighted and tumors were graded according to the ISUP system [1, 2]. Tumor quantitation was assessed as tumor load (TL), defined as the percentage of prostate involved by cancer. Specifically, our dedicated pathologist assessed tumor quantitation by visual estimation of all glass slides after every microscopically identifiable foci of carcinoma were circled with a marked pen, according to ISUP recommendations [8]. Surgical margins (SM) were stated positively when cancer invaded the inked surface of the specimen. In addition, they were classified as focal and non-focal according to linear extension [1, 2, 8]. Removed lymph nodes were counted and assessed for cancer invasion. Surgical specimens were staged by the 2017 version of the TNM system (8th edition) [1, 2].

### Evaluation of perioperative and oncological outcomes

Perioperative outcomes were assessed according to operating time, estimated intraoperative blood loss, nerve-sparing surgery, high and low-volume surgeons, and length of hospital stay (LOHS). Hospital readmission after discharge and postoperative complications were monitored for a period of at least 3 months and coded according to the Clavien-Dindo system [1, 2, 9]. Although patients were followed up according to the EAU guidelines (PSA measurement, disease-specific history, and digital rectal examination (DRE) every 6 months until 3 years and then annually), decisions for further treatment after surgery or in case of disease progression were taken in a multidisciplinary setting involving urologists, radiation oncologists, and oncologists to optimize recommendations with patients’ issues [1, 2].

### Evaluation of clinical and pathological factors predicting PCa progression

To verify our hypotheses, we investigated clinical and pathological factors predicting PCa progression. The primary endpoint was to assess the impact of prognostic factors on PCa progression, defined as biochemical recurrence/persistence and/or local recurrence and/or distant metastases. The secondary endpoint was to assess the association between clinical and pathological prognostic factors. Accordingly, clinical and pathological parameters were evaluated for study endpoints, excluding perioperative factors.

### Statistical methods

Continuous variables were measured by medians and interquartile ranges (IQR). Categorical factors were assessed for frequencies and rates (percentages). Time elapsed between surgery and the clinical outcome of interest (PCa progression) or last follow-up was measured as time first to event occurrence. Univariate and multivariate Cox proportional hazards models estimated the association of clinical and pathological factors with the risk of PCa progression, and hazard ratio (HR) and 95% confidence intervals (CI) were evaluated. The impact of significant prognostic factors on the median time to PCa progression was evaluated by the Kaplan-Meyer method (univariate analysis) and differences between groups by the Mantel-Cox log-rank test. Survival curves of PCa progression were generated. The binomial logistic regression model assessed associations between significant clinical and pathological factors (univariate and multivariate analysis), and risk of PCa progression. Finally, odds ratios (OR) and relative 95% CI were computed. The software used to perform the analysis was IBM-SPSS version 26. All tests were two-sided, and *p* < 0.05 was considered an index of statistical significance.

## Results

### General characteristics of the EAU high-risk population

Population general characteristics including 180 patients are shown in Table 1. Clinical disease factors were classified according to EAU guidelines. The median age was 66.5 [62–71] years. The younger and the older patients were 46 and 77 years old, respectively. Patients were more frequently classified as ASA score 2 (77.2%) and less frequently as ASA score 1 (7.1%). At clinical presentation, patients were more often younger than 70 years old (65.6%), and tumors were more often palpable (cT2/3; 68.3%) and high grade (ISUP 4/5; 61.1%), but less frequently with BPC ≥ 50% (43.9%) and PSA > 20 ng/mL (20.6%). In the surgical specimen, prostate tumors were more frequently high grade (ISUP 4/5; 80.6%) with an adverse stage (ECE, SVI) occurring in 45.6% of cases, including 30.6% with SVI. Overall, 60 patients (33.3%) had surgical margins involved by cancer. Extended pelvic lymph node dissection was performed in all cases, with PLNI detected in 47 (26.1%) subjects. The median (IQR) follow-up was 33.5 months (15–50 months). Adjuvant androgen deprivation therapy was given in 69 cases (38.3%) and RT was delivered in 61 (33.9%) subjects, with salvage purpose in 24 (13.3%). Deaths occurred in 2 patients (1.1%), none of them related to PCa progression.

**Table 1:** General characteristics of 180 prostate cancer (PCa) patients classified as high-risk class according to the European association of urology (EAU) prognostic system

Clinical factors	Statistics: median (IQR) or number (%)
Age (years)	66.5 [62–71]
< 70 years	118 (65.6)
≥ 70 years	62 (27.4)
BMI (kg/m^2^)	26.1 (24.3–28.6)
PV (mL)	40 (30–55)
ASA score 1	16 (8.9)
ASA score 2	139 (77.2)
ASA score 3	25 (13.9)
PSA ≤ 20 (ng/mL)	143 (79.4)
PSA > 20 (ng/mL)	37 (20.6)
BPC < 50%	101 (56.1)
BPC ≥ 50%	79 (43.9)
bISUP < 4	70 (38.9)
bISUP > 3	110 (61.1)
Tumors non palpable (cT1c)	57 (31.7)
Tumors palpable (cT2c)	98 (54.4)
Tumors palpable (cT3)	25 (13.9)
No enlarged pelvic lymph nodes (cN0)	131 (72.8)
Enalarged pelvic lymph nodes (cN1)	49 (27.2)
Pathological factors	
Prostate weight; PW (gr)	55 [43–65.6]
Tumor load; TL (%)	25 [15–40]
pISUP < 4	70 (38.9)
pISUP > 3	110 (61.1)
Organ confined disease; pT2	98 (54.4)
Extracapsular extension (ECE); pT3a	27 (15)
Seminal vesicle invasion (SVI); pT3b	55 (30.6)
Negative surgical margins; R0	120 (6.7)
Positive surgical margins; R1	60 (33.3)
No pelvic lymph node invasion (PLNI); pN0	133 (73.9)
PLNI; pN1	47 (26.1)
Number of counted lymph nodes; LN (*n*)	25 [20–32]

Continuous variables are reported as medians (IQR, interquartile ranges) and categorical factors as frequencies (percentages); *bISUP* biopsy ISUP, pISUP pathology ISUP, see materials and methods for abbreviations

### Clinical and pathological prognostic factors of PCa progression

**Fig. 1:**
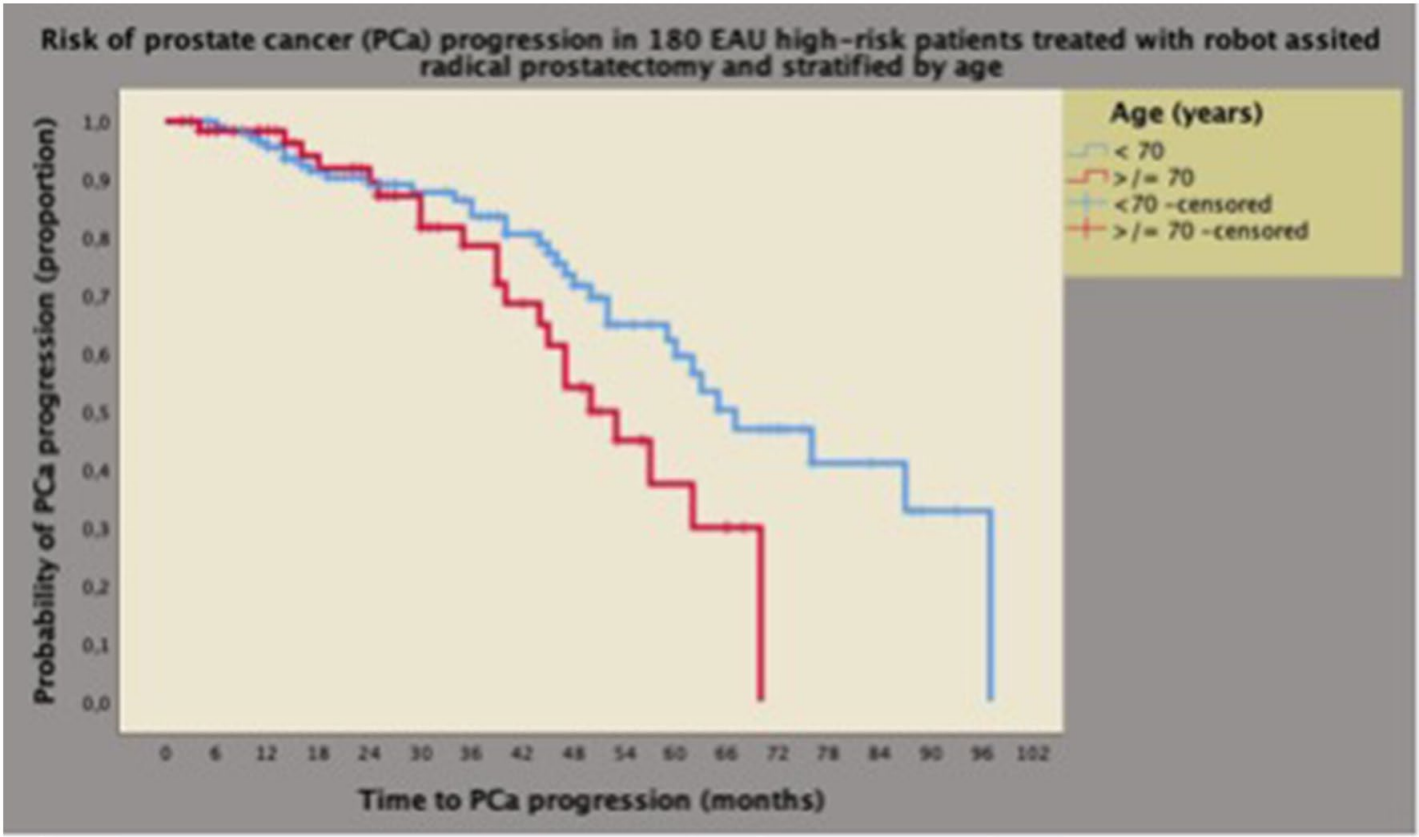
Kaplan–Meyer survival curves of PCa progression stratified by age groups in 180 high-risk patients classified according to the European Association of Urology (EAU) system. As shown, the median survival time of PCa progression was longer for younger cases (< 70 years) (67 months; 95% CI 53.3–80.6 months) than for older cases (50 months; 95% CI 41.2–58.7) and the difference was significant (Mantel-Cox log-rank test: *p* = 0.044). See also materials and methods, and results

**Fig. 2:**
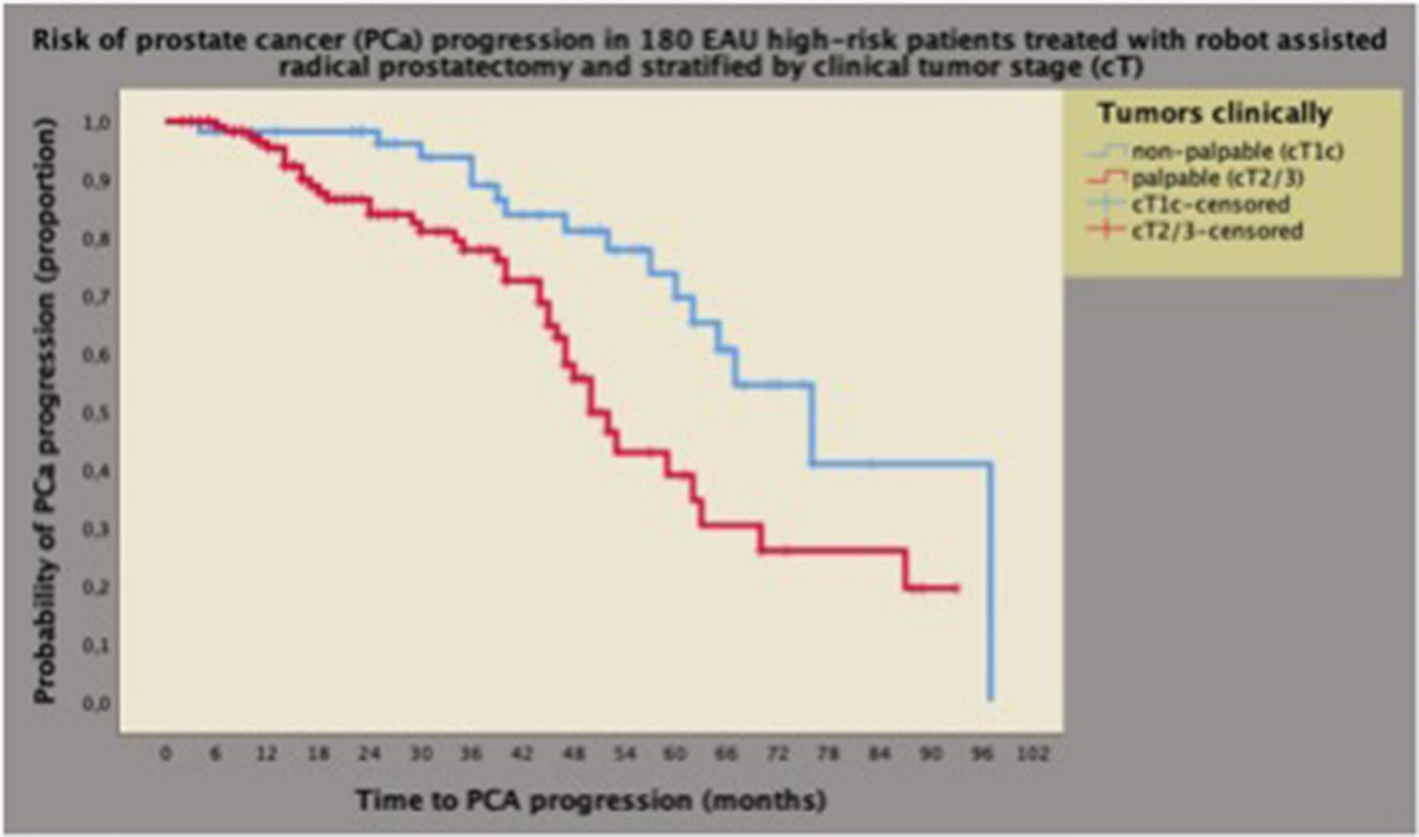
Univariate analysis of Kaplan–Meyer survival curves of PCa progression by prostate tumors at clinical presentation in 180 high-risk cases classified according to the European Association of Urology (EAU) system. Median survival time of PCa progression was longer for non-palpable tumors (76 months; 95% CI 60.6–91.3 months) than for the group presenting with palpable tumors (50 months; 95% CI 44.5–55.4 months), and the difference was significant (Mantel-Cox log-rank test: *p* = 0.004). See also materials and methods, and results

**Fig. 3:**
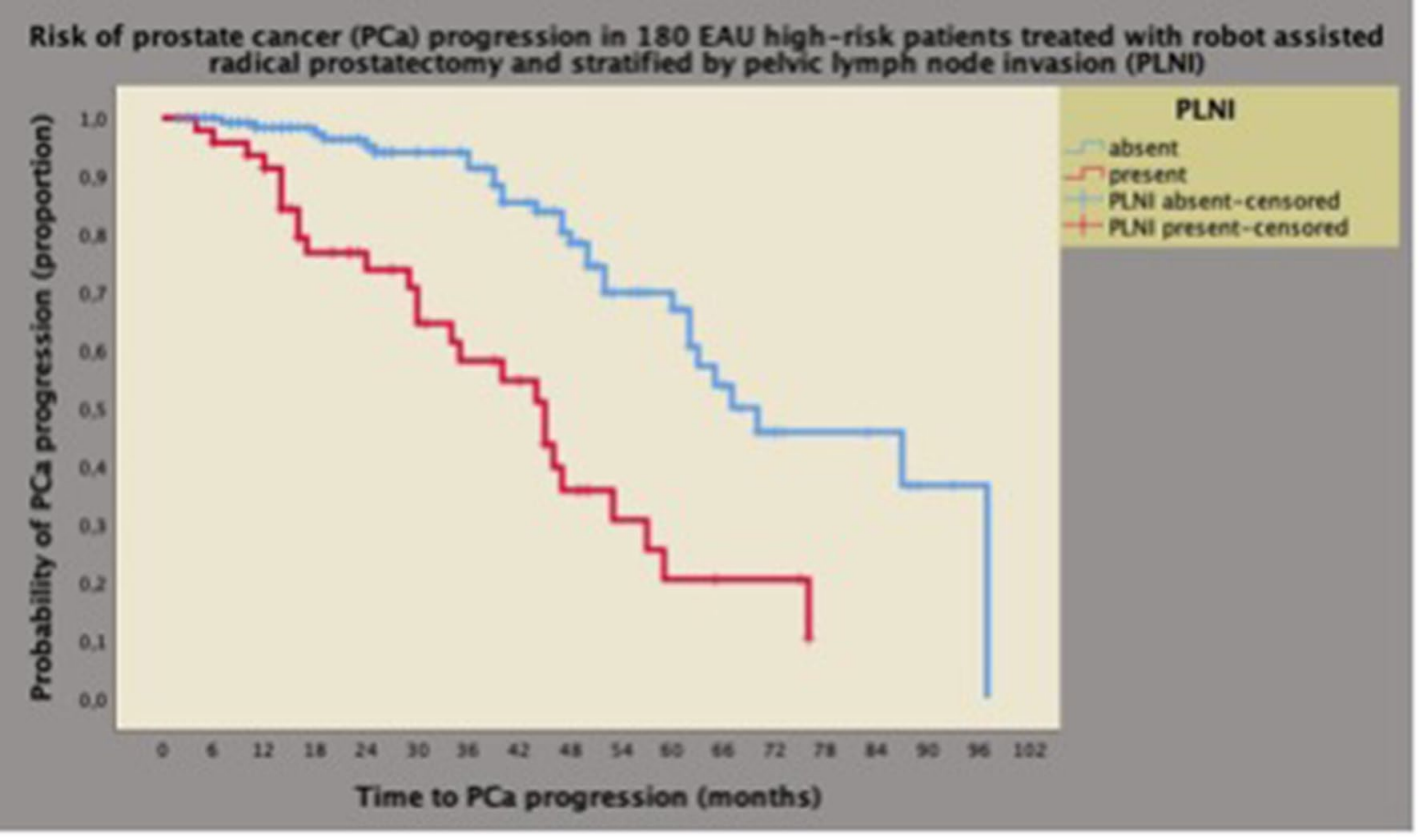
Univariate analysis of Kaplan–Meyer survival curves of PCa progression stratified by pelvic lymph node invasion (PLNI) in 180 high-risk patients classified according to the European Association of Urology (EAU) system. As shown, the median survival time of PCa progression was longer for patients without PLNI (70 months; 95% CI 50.5–89.4 months) than for cases with PLNI (45 months; 95% CI 32.9–57.0), and the difference was significant (Mantel-Cox log-rank test: *p* < 0.0001). See also materials and methods, and results

As shown in Table 2, disease progression occurred in 55 patients (30.6%), who were more likely to present with advanced age and palpable tumors, as well as unfavorable pathology features, including high tumor grade (ISUP 4/5) and stage (SVI), positive surgical margins and PLNI. However, PCa progression was independently predicted by advanced age, tumor palpability, and by PLNI. Notably, other adverse clinical factors (PSA > 20 ng/mL, BPC ≥ 50%, ISUP 4/5) showed no association, and other pathological factors lost significance on multivariate analysis. Accordingly, the risk of PCa progression increased for advanced age, clinically palpable tumors, and tumors spread to pelvic lymph nodes. As a result, median (95% CI) PCa survival curves were progressively unfavorable for these prognostic factors, as shown in Figs. 1, 2 and 3, which provide detailed information on Kaplan-Meyer survival curves.

**Table 2:** Risk of prostate cancer (PCa) progression by clinical and pathological factors in 180 EAU high-risk patients treated with robot-assisted radical prostatectomy (RARP) and extended pelvic lymph node dissection (ePLND)

Number (%)	No PCa progression	PCa progression	Univariate analysis		Multivariate analysis	
	125 (69.4)	55 (30.6)	HR (95% CI)	*p* value	HR (95% CI)	*p* value
Age ≥ 70 years	41 (32.8)	21 (38.2)	1.759 (1.004–3.083)	0.048	2.183 (1.089–4.377)	0.028
BMI (kg/m^2^)	25.8 (24.4–28.7)	26.2 (23.7–28.4)	0.998 (0.913–1.068)	0.755	0.940 (0.860–1.028)	0.176
PV (mL)	40 (30–55)	39 (25–58)	1.007 (0.992–1.022)	0.371	1.000 (0.973–1.027)	0.973
PSA > 20 (ng/mL)	18 (14.4)	19 (34.5)	1.519 (0.865–2.667)	0.145	1.342 (0.666–2.705)	0.411
BPC ≥ 50%	50 (40)	29 (52.7)	1.515 (0.885–2.593)	0.130	0.851 (0.432–1.675)	0.640
bISUP > 3	74 (59.2)	36 (65.5)	0.872 (0.496–1.535)	0.636	0.524 (0.238–1.155)	0.109
Tumors palpable (cT2/3)	84 (76.2)	39 (70.9)	2.393 (1.304–4.391)	0.005	3.113 (1.499–6.465)	0.002
cN1	33 (26.4)	16 (29.1)	1.414 (0.787–2.541)	0.247	1.534 (0.735–3.199)	0.254
PW (gr)	52 (43–66)	57 (43–65.8)	1,009 (0.995–1.022)	0.205	0.999 (0.975–1.024)	0.932
TL (%)	20 (10–40)	40 (20–60)	1,018 (1.006–1.029)	0.002	1.012 (0.996–1.028)	0.159
pISUP > 3	65 (52)	45 (81.8)	2,512 (1.262–5.006)	0.018	1.671 (0.688–4.444)	0.324
ECE	21 (16.8)	6 (10.9)	1.240 (0.490–3.135)	0.650	0.987 (0.353–2.757)	0.980
SVI	25 (20)	30 (54.5)	2.661 (1.479–4.788)	0.001	1.264 (0.546–2.928)	0.584
R1	36 (28.8)	24 (43.6)	1,953 (1.135–3.359)	0.016	1.104 (0.545–2.238)	0.784
LN (*n*)	25 (20–33)	25 (19–32)	0.999 (0.971–1.028)	0.950	1.012 (0.979–1.016)	0.494
PLNI (pN1)	36 (28.8)	24 (43.6)	3.532 (2.055–6.071)	< 0.0001	2.945 (1.441–6.018)	0.003

Continuous variables are reported as medians (IQR, interquartile ranges) and categorical factors as frequencies (percentages); *HR* hazard ratio; *CI* confidence interval; see also Table 1, materials and methods for abbreviations. Univariate and multivariate analyses performed with Cox proportional hazard models

### Predictors of PLNI in EAU high-risk PCa

Table 3 shows the analysis of clinical factors predicting PLNI in high-risk patients undergoing anatomic staging with ePLND. As shown, adverse clinical factors, including BPC ≥ 50%, PSA > 20 ng/mL, and ISUP 4/5 were associated with the risk of PLNI. On multivariate analysis, after adjustment for the number of lymph nodes counted, patients were more likely to have PLNI for tumors with adverse PSA (PSA > 20 ng/mL), as well as for large (BPC ≥ 50%,) and undifferentiated (ISUP 4/5) tumors. Further details are shown in the referred Table.

**Table 3:** Clinical predictors of pelvic lymph node invasion (PLNI) in 180 EAU high risk patients treated with robot assisted radical prostatectomy (RARP) and extended pelvic lymph node dissection (ePLND)

Number (%)	No PLNI	PLNI	Univariate analysis	*p* value	Multivariate analysis*	*p* value
	133 (73.9)	47 (26.1)	OR (95% CI)		OR (95% CI)	
Age ≥ 70 years	42 (31.6)	20 (42.6)	1.005 (0.810–3.181)	0.175	1.330 (0.601–2.942)	0.482
BMI (kg/m^2^)	25.7 (24.3–28.7)	26.3 (23.5–28.6)	1.005 (0.913–1.106)	0.916	0.944 (0.846–1.054)	0.306
PV (mL)	40 (30–52)	45 (33.1–70)	1.012 (0.995–1.029)	0.178	1.016 (0.996–1.037)	0.118
PSA > 20 (ng/mL)	24 (18)	13 (27.7)	1.737 (0.798–3.777)	0.164	3.032 (1.181–7.787)	0.021
BPC ≥ 50%	51 (38.3)	28 (59.6)	2.369 (1.201–4.674)	0.013	2.361 (1.108–5.033)	0.026
bISUP > 3	72 (54.1)	38 (80.9)	3.577 (1.603–7.982)	0.002	5.065 (1.979–12.963)	0.001
cT2/3	90 (67.7)	33 (70.2)	1.126 (0.547–2.321)	0.747	1.552 (0.666–3.618)	0.309
cN1	37 (27.8)	12 (25.5)	0.890 (0.417–1.897)	0.762	1.698 (0.698–4.129)	0.243
LN (*n*)	24 (19–31)	29 (20–34)	1.030 (0.996–1.066)	0.083	1.032 (0.994–1.072)	0.103

Continuous variables are reported as medians (IQR, interquartile ranges) and categorical factors as frequencies (percentages); *OR* odds ratio (binomial logistic regression model); *CI* confidence interval

*Adjusted for the number of counted lymph nodes; see also Table 1, materials and methods for abbreviations

### The impact of older age on diagnosis and progression of high-risk PCa

Table 4 illustrates associations of age with other clinical variables at diagnosis and summarizes the impact of age on disease progression in high-risk PCa. As shown, older patients were less likely to have palpable tumors (cT2/3), but more likely to have high-grade tumors (ISUP 4/5). As a result, older patients were more likely to have disease progression, regardless of the association of palpable tumors or PLNI. Further details are reported in the above Table and Figs. 1, 2 and 3. We also assessed that adverse clinical tumor stage (cT2/3) was independently and inversely associated with PSA ≤ 20 ng/mL (OR = 0.398; 95% CI 0.185–0.858; *p* = 0.019) and younger age (age < 70 years); consequently, younger patients were less likely to have palpable tumors and PSA > 20 ng/mL (data not shown in Table 4).

**Table 4:** The role of age on diagnosis and progression in 180 EAU high-risk patients treated with robot-assisted radical prostatectomy and extended pelvic lymph node dissection

Patients	Age < 70 years	Age ≥ 70 years	*p* value
	118 (65.6%)	62 (27.4%)	
Diagnosis of high-risk Pca			
Tumors non-palpable (cT1c)	31 (26.3)	26 (41.9)	
Tumors palpable (cT2/3)	87 (73.7)	36 (58.1)	
Unadjusted OR (95% CI)	Ref	0.493 (0.258–0.945)	0.033
Adjusted OR (95% CI) (*)	Ref	0,424 (0.209–0.858)	0.017
ISUP < 4	55 (46.6)	15 (24.2)	
ISUP > 3	63 (53.4)	47 (75.8)	
Unadjusted OR (95% CI)	Ref	2.735 (1.379–5.424)	0.004
Adjusted OR (95% CI)*	Ref	2.509 (1.162–5.419)	0.019
Risk of PCa progression			
No progression	84 (67.2)	34 (61.8)	
Disease progression	41 (38.8)	21 (38.2)	
Unadjusted HR (95% CI)	Ref	1.759 (1.004–3.083)	0.048
Adjusted HR (95% CI)**	Ref	2.095 (1.062–4.136)	0.033

*OR* Odds ratio (binomial logistic regression model); *HR* hazard ratio (Cox proportional hazard models); *CI* confidence interval

*Model adjusted for remaining clinical factors included in Table 2

**Model adjusted for all clinical and pathological factors included in Table 2

## Discussion

Oncological outcomes after RARP in high-risk patients depend on the primary endpoints assessed. These include biochemical recurrence, disease progression, overall survival, and cancer-specific survival. While the former is the easiest to assess, the latter is more significant but requires longer follow-up. Contemporary literature confirms that early biochemical recurrence and disease progression is predicted by well-known standard factors that define the inclusion criteria for high-risk patients; consequently, as the number of preoperative risk factors increases, the risk of disease progression increases [10–12]. When an appropriate anatomical staging of pelvic nodes has been performed, PLNI is an independent predictor of biochemical recurrence and disease progression [13, 14]. Our study shows that, in a population of high-risk PCa undergoing surgery, the strongest independent prognostic factors of disease progression were PLNI, which was clinically predicted by well-validated prognostic factors including PSA > 20 ng/mL, adverse tumor stage (cT2/3) and grade (ISUP 4/5). Notably, the number of lymph nodes counted had no impact on the detection of PLND, thus confirming that an ePLND is mandatory when treating with primary surgery this category of patients in need of accurate anatomic staging. So far, as the number of prognostic factors has increased, the risk of detecting PLNI has increased and the prognosis has worsened. In contrast, as the number of clinical predictors decreased, high-risk patients were less likely to have PLNI and thus a more favorable prognosis.

Age is an important factor to consider when dealing with high-risk PCa patients, which may include middle- and advanced-aged groups. Consequently, assessing prognostic factors in age groups is important to reduce mortality rates related to metastases and recurrences. Nomograms predicting cancer-specific survival (CSS) and overall survival (OS) show good accuracy and reliability [15]. However, advanced age has not been shown to be an independent predictor of disease progression [10–14]. In tertiary referral centers, clinically localized PCa in elderly patients is increasingly treated with RARP, ranging from 7.5% to 26.8%. Accordingly, a previous study showed that senior cases comprised nearly one-third (27%) of the PCa population treated with RARP. Although elderly patients were more likely to belong to the EAU high-risk class, advanced age was not an independent predictor of disease progression in the overall patient population [16]. In this study, we demonstrated that advanced age and palpable tumors were clinically independent predictors of PCa progression in the high-risk population. According to our results, elderly patients are more likely to have PCa progression, although they are less likely to have palpable tumors associated with lower PSA levels. Elderly patients with other adverse clinical factors (PSA > 20 ng/mL, ISUP 4/5, and cT2/3) represent the worst clinical prognostic group for PLNI risk. In contrast, younger patients (age < 70 years) are less likely to have disease progression and PLNI because they have non-palpable tumors and lower PSA levels. So far, the age group has been shown to be an important prognostic factor in the diagnosis of high-risk PCa. This seems to be a novelty for the literature dealing with high-risk PCa patients treated with RARP and ePLND. However, confirmatory studies are needed.

Prognostic factors of disease progression, have implications in clinical practice when treating high-risk PCa patients and in assessing oncological outcomes. Accordingly, as the number of prognostic factors rises, the risk of PCa progression increases. Consequently, age may represent an additional clinical prognostic factor when evaluating this group of patients [1, 2, 10–16]. The results of our study provide important information for clinicians (urologists and radiation oncologists) when counseling high-risk PCa patients in which age is an additional prognostic factor for their classification. Older patients are more likely to present clinically with unfavorable tumor stage and grade, which are also predictors of PLNI, and have disease progression, representing an unfavorable prognostic subgroup. Conversely, younger patients with non-palpable tumors, associated with lower PSA levels and more favorable grade groups, are less likely to have PLNI and to experience PCa progression, representing a more favorable prognostic group. This information will help clinicians counsel patients and decide on further action before and after combined primary treatments.

Explanations are needed to interpret the results of our study, in which older patients (age ≥ 70 years) were more likely to have an unfavorable tumor grade and stage, both predictive of PLNI, as well as to have PCa progression. Theoretically, high-grade palpable tumors expressing lower PSA levels are more undifferentiated and therefore more likely to progress locally and invade pelvic loco-regional lymph nodes. In addition, these aggressive tumors develop and progress in a microenvironment compromised by aging. This exposes cancer cells to progressively acquiring lethal genetic mutations, a typical pattern of progression of PCa, and to develop in a compromised immune system. In addition, impaired endogenous testosterone levels, which are more pronounced in elder patients, could play a major role in the natural history of PCa, an endocrine-dependent tumor [17–19]. These hypotheses need to be verified by controlled studies.

Our study has several limitations. It was retrospective and involved a single center. It could suffer from a possible selection bias because 242 of the 1143 patients treated with RARP during the considered period were not included in the data collection for missing follow-up. mp-MRI and molecular and/or genetic tests were not evaluated because they were not available in all cases. We did not evaluate overall and cancer-specific survival because of the limited number of such events, and we did not calculate the percentage of cancer involving each biopsy core because of a lack of data in some cases. Finally, the percentage of Gleason pattern 4 was not evaluated in biopsy-positive cores.

However, there are strengths in our study. The primary outcome was assessed at disease progression, which is a stronger endpoint than biochemical recurrence alone. Procedures were performed by low and high-volume surgeons who did not bias staging results, reflecting actual practice in tertiary referral centers. The length of follow-up was appropriate to evaluate the primary endpoint. Finally, all surgical specimens were specifically evaluated by our dedicated pathologist, and ePLND was appropriate because of the inclusion of an average of 25 counted lymph nodes in the surgical specimen.

## Conclusions

In a high-risk PCa population treated with RARP and ePLND, disease progression was predicted clinically by advanced age and by tumor palpability, pathologically by PLNI, which was predicted by standard clinical prognostic factors. Therefore, the prognosis worsened with increasing age in elderly patients, who were more likely to have palpable and undifferentiated tumors. In high-risk PCA, elderly patients represent an unfavorable age group that needs extensive counseling for appropriate and personalized management decisions.

## Data Availability

The data presented in this study are available on request from the corresponding author. The data are not publicly available due to ethical reasons.
